# Characterizing Features of Human Circulating B Cells Carrying CLL-Like Stereotyped Immunoglobulin Rearrangements

**DOI:** 10.3389/fonc.2022.894419

**Published:** 2022-06-23

**Authors:** Davide Bagnara, Monica Colombo, Daniele Reverberi, Serena Matis, Rosanna Massara, Niccolò Cardente, Gianluca Ubezio, Vanessa Agostini, Luca Agnelli, Antonino Neri, Martina Cardillo, Stefano Vergani, Fabio Ghiotto, Andrea Nicola Mazzarello, Fortunato Morabito, Giovanna Cutrona, Manlio Ferrarini, Franco Fais

**Affiliations:** ^1^ Department of Experimental Medicine, University of Genoa, Genoa, Italy; ^2^ Molecular Pathology, IRCCS Ospedale Policlinico San Martino, Genoa, Italy; ^3^ Transfusion Centre, IRCCS Ospedale Policlinico San Martino, Genoa, Italy; ^4^ Department of Pathology, IRCCS Istituto Nazionale dei Tumori, Milan, Italy; ^5^ Department of Oncology and Hemato-oncology, University of Milan, Milan, Italy; ^6^ Scientific Directorate, Azienza Unità Sanitaria Locale (USL)-IRCCS di Reggio Emilia, Reggio Emilia, Italy; ^7^ Karches Center for Oncology Research, The Feinstein Institute for Medical Research, Manhasset, NY, United States; ^8^ Developmental Immunology Unit, Division of Molecular Hematology, Department of Laboratory Medicine, Lund Stem Cell Center, Lund University, Lund, Sweden; ^9^ Hematology and Bone Marrow Transplant Unit, Hemato-Oncology Department, Augusta Victoria Hospital, East Jerusalem, Israel; ^10^ Biothecnology Research Unit, AO of Cosenza, Cosenza, Italy

**Keywords:** chronic lymphocytic leukemia (CLL), immunoglobulin repertoire, B-cells, CD5, IGHV somatic mutations, Ig light chain, CLL stereotyped BCR

## Abstract

Chronic Lymphocytic Leukemia (CLL) is characterized by the accumulation of monoclonal CD5^+^ B cells with low surface immunoglobulins (IG). About 40% of CLL clones utilize quasi-identical B cell receptors, defined as stereotyped BCR. CLL-like stereotyped-IG rearrangements are present in normal B cells as a part of the public IG repertoire. In this study, we collected details on the representation and features of CLL-like stereotyped-IG in the IGH repertoire of B-cell subpopulations purified from the peripheral blood of nine healthy donors. The B-cell subpopulations were also fractioned according to the expression of surface CD5 molecules and IG light chain, IGκ and IGλ. IG rearrangements, obtained by high throughput sequencing, were scanned for the presence of CLL-like stereotyped-IG. CLL-like stereotyped-IG did not accumulate preferentially in the CD5^+^ B cells, nor in specific B-cell subpopulations or the CD5^+^ cell fraction thereof, and their distribution was not restricted to a single IG light chain type. CLL-like stereotyped-IG shared with the corresponding CLL stereotype rearrangements the IGHV mutational status. Instead, for other features such as IGHV genes and frequency, CLL stereotyped-IGs presented a CLL-like subset specific behavior which could, or could not, be consistent with CLL stereotyped-IGs. Therefore, as opposed to the immuno-phenotype, the features of the CLL stereotyped-IG repertoire suggest a CLL stereotyped subset-specific ontogeny. Overall, these findings suggest that the immune-genotype can provide essential details in tracking and defining the CLL cell of origin.

## Introduction

Chronic Lymphocytic Leukemia (CLL) is characterized by the accumulation of monoclonal B lymphocytes expressing CD5, CD23, and low surface immunoglobulin in blood, bone marrow, and lymphoid tissues (1, 2). Analyses of many CLL clones demonstrated that the IG gene rearrangements encoding the CLL B cell receptor (BCR) exhibit a striking skewed use of IGHV genes resulting in an IG repertoire different from that of normal B lymphocytes (3–5). Moreover, despite the enormous diversity potentially generated by the recombination of IGHV-IGHD-IGHJ genes, up to 40% of the CLL clones (6, 7) exhibit highly similar stereotyped BCR, which has led to the categorization of the CLL clones with stereotyped BCR into subsets based on their similarities. Although several hundred CLL subsets have been identified, those most frequently encountered, defined as “major subsets,” are limited in numbers. Stereotyped BCRs are determined based on the VH CDR3 features of at least 50% of amino acids identity and 70% of amino acid motif similarities, identical VH CDR3 length and location of a shared pattern(s) among sequences of the same group (6, 8), and the use of IGHV genes belonging to the same phylogenetic clan. IGHV clans are IGHV family genes with structural similarities (9). Conceptually, the stereotyped gene rearrangements should be considered part of the public IG repertoire because different individuals share them (10). In addition, CLL clones of the same stereotyped subsets show IG light chain restrictions (e.g., #1, #2, #4, #6, #8, #64b, and #99) (6, 11–13) and IGKV-IGKJ/IGLV-IGLJ gene rearrangements presenting stereotypy features similar to those of IGHV rearrangements with limitations in IG light chain gene usage and VL CDR3 composition.

The above observations support the notion of a role for BCR stimulation in CLL ontogeny (14–16); moreover, the results of therapies with inhibitors of BCR-associated kinases suggest that stimulation *via* BCR may be critical for the survival/proliferation of CLL cells in full-blown leukemia (17).

Previous studies have identified IGHV-IGHD-IGHJ rearrangements sharing features with that characteristic of CLL subsets in splenic and circulating B cells from normal subjects (18–20). These rearrangements, from now onward defined as CLL-like stereotyped-IG or CLS-IG, can be observed in different B cell subpopulations, even though they accumulate in the CD5^+^ B-cells (20, 21).

This study used high-throughput sequencing technology on peripheral blood B cell subsets to elucidate CLS-IG’s features and cellular distribution. For this purpose, the cells, separated into defined subsets, were also fractionated according to CD5 or IG light chain expression. The data obtained provide a new perspective for interpreting the origin of CLL cell repertoire and possibly for disease ontogeny.

## Materials and Methods

### Samples

Peripheral blood cells were obtained from the leukopak of anonymous blood donors (nine donors aged 55 to 64 years old) at the San Martino Hospital Blood Center presentation. Each leukopak is derived from ~500ml of blood. B cells were enriched with RosetteSep Human B Cell Enrichment Cocktail (Stemcell Technologies, Vancouver, Canada), obtaining on average 42x10^6^ B cells per donor (24x10^6^ to 60x10^6^) (**Supplementary Table S1**).

### Isolation of PB B Cell Subpopulations and Fractionation of CD5^+^, CD5^-^ B Cells, and IGκ and IGλ

**Figure 1:**
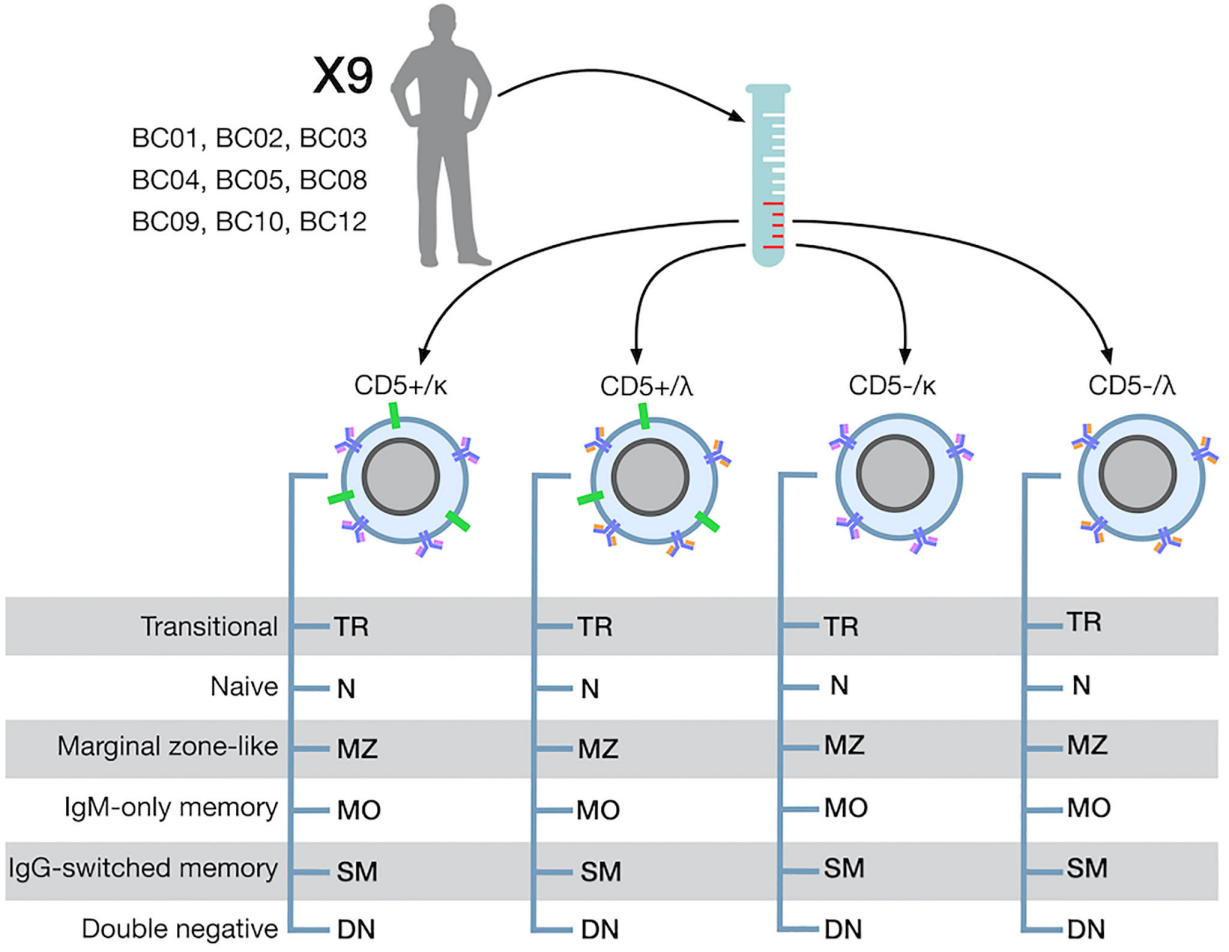
B-cell separation strategy.

B cells enriched cell fractions were stained with the following combination of mAbs: anti-IgD Alexa Vio770 (BioLegend, San Diego, CA, USA); anti-IgM PerCP_Cy5.5, anti-CD27 PE-CF594, anti-CD38 PE-Cy7, anti-CD24 Alexa Fluor 647 and anti-CD5 BV 421, anti-IGκ FITC, anti-IGλ PE, anti-IgA VioGreen (BD). B cell subsets were isolated by FACS sorting (FACSAria, Becton Dickinson, Franklin Lakes, NJ, USA) after depleting IgA^+^ and dead cells with a two-step sorting approach: 1) a four-way pre-sort with yield setting was used to separate enriched B cells into IGκ^+^/CD5^+^, IGκ^+^/CD5^-^, IGλ^+^/CD5^+,^and IGλ^+^/CD5^-^B cells; 2) each of the above cell fractions were then sorted into six main B cell subpopulations (after excluding CD38^high^CD24^-^ plasmablasts): CD24^high^CD38^high^ transitional (TR), IgD^high^IgM^+^CD38^-^CD27^-^ naive (N), IgD^low^ IgM^+^CD38^-^CD27^+^ marginal zone-like (MZ), IgM^+^IgD^-^CD38^-^CD27^+^ IgM-only memory (MO), IgM^-^IgD^-^CD38^-^CD27^+^ switch-memory (SM), and IgM^-^IgD^-^CD38^-^CD27^-^ double negative (DN) B cells. See also **Figure 1** and **Supplementary Figure S1** for details.

### Library Preparation and Sequencing

IGH sequencing and analysis were performed as previously reported in detail (22). Briefly, the library was prepared from mRNA with a multiplex approach with IGHV-specific forward primers on the leader sequence and reverse primers on the constant region. The primer set was kindly provided by TIB Molbiol srl (Genoa, Italy). The amplicons obtained included the entire IGHV-IGHD-IGHJ gene and enough constant region to assign the isotype; UMIs (14 to 16 nucleotides) were inserted during ds-cDNA synthesis. The libraries were indexed with Illumina Nextera XT V2 kit (Illumina, San Diego, CA, USA) and sequenced on Illumina MiSeq (MiSeq V3, 2x300 kit, Illumina). DNA sequences were deposited on the NCBI Sequence Read Archive (SRA) portal with BioProject ID: PRJNA807871.

### Bioinformatics Analysis

Raw reads were processed with a custom-built workflow using pRESTO (23) as previously described (20); processed sequences were then annotated by IMGT/HighV-QUEST (24). Only productive rearrangements derived from the consensus of two or more raw reads without N nucleotide passed the quality filter. ChangeO (25) was used to define and annotate clonotypes as sequences with identical amino acid VH CDR3 sequence using the same IGHV gene and IGHJ gene.

### CLL Subsets Assignment

To identify CLL-like stereotyped sequences (CLS-IG), we first selected the sequences in our database consistent with the core features (IGHV clan, IGHV mutational status, and VH CDR3 length) (26) of each of the 19 major CLL stereotyped subsets and then submitted them to ARResT/AssignSubsets (26) for the assignment. Sequences assigned to CLL stereotyped subsets with a confidence “average” or higher were considered CLS-IG. Sequences not assigned to CLL stereotyped subsets were defined as non-CLS-IG. The entire analysis was then performed at the clonal level, i.e., for each clonotype, a single representative sequence was considered.

To identify VH CDR3 aa sequences consistent with a CLL stereotyped subset but with the reverse (r) mutational status compared to the core feature of CLL stereotypies (rCLS-IG), we re-submitted to aRResT the IGHV-IGHD-IGHJ rearrangements sequences in which the original IGHV gene region was replaced with one with the opposite mutational status, generating an *in silico* chimeric sequence.

### Statistics

Statistical analyses were performed in R. Paired Wilcoxon tests or binomial test with Bonferroni correction applied to assess differences in CLS-IG or VH gene frequency (*p ≤ 0.05, **p ≤ 0.01, ***p ≤ 0.001). The paired Wilcoxon test was calculated only if three or more donors presented data for both points. CLS-IGs’ frequency was calculated only when the number of CLS-IG sequences in the group was two or more. Frequencies obtained from only one CLS-IG sequence within a group were not considered informative and therefore not plotted unless specified.

## Results

### Identification of CLL-Like Stereotyped-IGs in B-Cell Subpopulations

Peripheral blood B cell samples from each of nine donors were separated into 24 phenotypically distinct cell fractions. First, B cells were divided into four fractions according to the presence or absence of surface CD5 and surface IG light chain expression (IGκ or IGλ). From each of the four B cell fractions, six different B-cell subpopulations (B-subset) were isolated: transitional (TR), naive (N), marginal zone-like (MZ), IgM only memory (MO), IgG switched memory (SM), and double-negative (DN); see **Figure 1**, Methods and **Supplementary Figure S1** for phenotypes and details. After quality filtering, a total of 2,679,224 productive, unique IGHV-IGHD-IGHJ sequences were obtained from 8,043,500 sorted B cells. Curated sequences were subsequently clustered into 2,184,656 clonotypes (detailed in **Supplementary Table S2**), of which 1754 (0.08%) were assigned to one of the major CLL stereotyped subsets (CLL-subset) with ARResT/AssignSubsets (26) (as detailed in Methods) and defined as CLL-like stereotypes-IG (CLS-IG) (detailed in **Supplementary Table S3**). Clonal families were used as references for the entire analysis.

### Correlation of the Higher CLL-Like Stereotyped-IG Representation in CD5^+^ B Cells With an Asymmetrical Distribution of U and M IGHV Rearrangements in CD5^+^ and CD5^-^ B Cells

**Figure 2:**
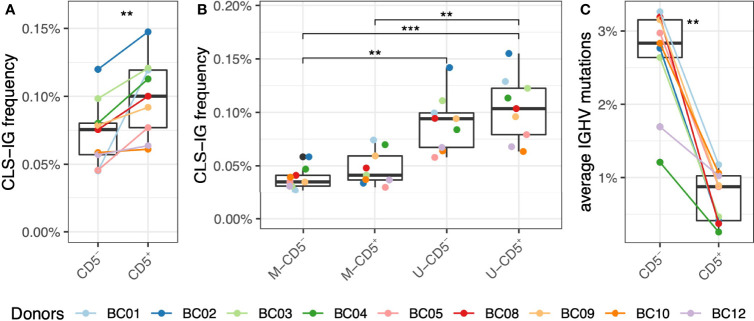
Frequency of CLS-IG in CD5^+^ and CD5^-^ B cells. **(A)** CLS-IG frequency in CD5^+^ and CD5^-^ B cells analyzed in bulk. **(B)** Frequency of CLS-IG in the M and U IGHV rearrangements of CD5^+^ and CD5^-^ B cells analyzed separately. **(C)** IGHV genes mutation distribution in CD5^+^ and CD5^-^ B cells. Paired Wilcoxon test was performed, only significant statistics are shown (**p ≤ 0.01, ***p ≤ 0.001).

First, sequences from all CD5^+^ or CD5^-^ subpopulations respectively were pooled and analyzed for the presence of CSL-IG to see whether the CSL-IG were predominant in the CD5^+^ cell fraction, and we found that the proportion of CLS-IG was significantly higher in CD5^+^ than in CD5^-^ B cells (**Figure 2A**). However, when the IGHV gene rearrangements were separated into mutated (≥2% IGHV gene mutations, M-IG) and unmutated (<2% mutations, U-IG), there was no difference in the frequency of CLS-IG between CD5^+^ and CD5^-^ B cells within a single mutational status group (**Figure 2B**). Furthermore, U-IG clonotypes had more CLS-IG than M-IG clonotypes in both CD5^+^ and the CD5^-^ populations. When looking at the average IGHV mutation frequency, the CD5^+^ clonotypes appear to be enriched in U-IG, contrary to CD5^-^ clonotypes enriched in M-IG (**Figure 2C**).

### Presence of CLL-Like Stereotyped-IG in B-Cell Subpopulations

**Figure 3:**
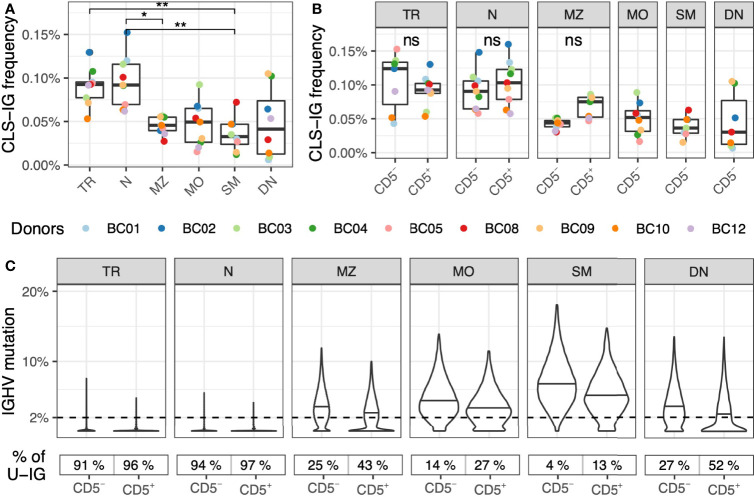
Frequency of CLS-IG in circulating B-subset. **(A)** Frequency of CLS-IG in B-subset **(B)**. Frequency of CLS-IG in the same B-subset fractionated further for CD5^+^ and CD5^–^ B cells. Paired Wilcoxon test was performed, only significant statistics are shown (*p ≤ 0.05, **p ≤ 0.01; ns, not significant). CLS-IG identified in the CD5^+^ fraction of MZ, MO, SM, and DN B cells were insufficient for statistical analysis. **(C)** IGHV genes mutation distribution in the B cell subsets-sub divided by CD5 expression. The percentage of unmutated IGs (< 2%) in each B cell population is shown at the bottom of the figure.

CLS-IG were found in all B-subsets, although in different proportions (**Figure 3A**); i.e., N and TR B cells had a significantly higher CLS-IG representation (0.09%) than MZ (0.05%) and SM (0.03%) B cells. N and TR B cells also had the highest U-IG sequences (**Figure 3C**). Further fractionation of each B-subset into CD5^+^ and CD5^-^ cells did not show a significant predominance of CLS-IG in any of the CD5^+^ cell fractions (**Figure 3B**). In most cases, SM, DN, and MO B-subsets presented none or just one CLS-IG per donor. It must be noted that these B-subsets have the lower frequency of CD5^+^ cells and, therefore, the least IGH sequences (**Supplementary Table S2**).

### Different Frequencies of CLL-Like Stereotyped-IG Subsets in Normal B Cells and CLL Clones

**Figure 4:**
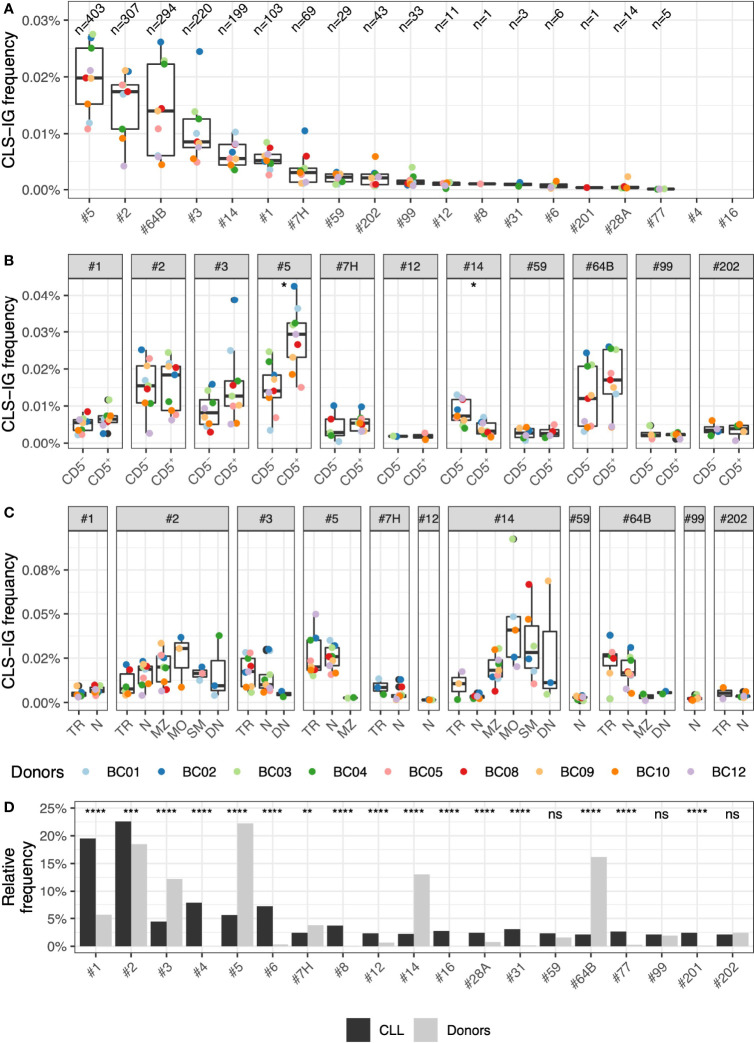
Frequency of CLS-IG subsets. **(A)** Frequency of CLS-IGs for individual CLL subsets in healthy donors among all IGHV-IGHD-IGHJ clonotypes. **(B)** Frequency of CLS-IGs for each CLL subset in CD5^+^ and CD5^-^ B cells. Frequency data derived from only one donor was not considered informative and not plotted. Paired Wilcoxon test was performed, only significant statistics are shown (*p ≤ 0.05). **(C)** Frequency of CLS-IGs for each CLL subset in B cell subsets. Frequency data derived from only one donor was not considered informative and not plotted. **(D)** Frequency of CLS-IGs for individual CLL subsets among all CLS-IGCLL subset frequency of CLS-IGs relative frequency compared with CLL stereotyped clones (6). Binomial test was performed, only significant statistics are shown (*p ≤ 0.05, **p ≤ 0.01, ***p ≤ 0.001, ****p ≤ 0.0001; ns, not significant).

Overall, the median frequency of CLS-IGs clonotypes of individual CLL subsets was within the range of 0.019% to undetectable in circulating B cells (**Figure 4A**). Subset #5 was the most represented (403 clonotypes, 0.019%), followed by subset #2 (307 clonotypes, 0.017%), subset #64B (294 clonotypes, 0.014%), subset #3 (220 clonotypes, 0.008%), subset #14 (201 clonotypes, 0.006%), and subset #1 (103 clonotypes, 0.005%), whereas other CLL subsets were represented at a lower level. CLS-IG frequency was comparable between CD5^+^ and CD5^-^ B cells for every CLL subset except subset #5 where CD5^+^ presented statistically more CLS-IGs than CD5^-^, and subset #14, where CD5^+^ presented statistically fewer CLS-IGs than CD5^+^ (**Figure 4B**). For CLL subsets with U-IGs, we identified CLS-IG consistently only in N and TR B-subsets, whereas for CLL subsets with M-IGs (#2 and #14), CLS-IG were reproducibly detected also in MZ, MO, SM, and DN B-subsets (**Figure 4C**).

The CLS-IG frequency among the CLL subsets in our dataset was compared to CLL stereotyped-IGs reported in the study by Agathangelidis et al. (6). While the distribution of most CLL subsets did present statistically significant differences between CLL stereotyped IG and CLS-IG, only a few differed substantially (**Figure 4D**). Subset #1, which is relatively frequent in CLL, was much less represented in the CLS-IG of peripheral blood, whereas subsets #5, #14, and #64B, the most abundant in normal B cells, were much less frequent in the CLL dataset. Other subsets were absent (#4 and #16) or rarely observed (#6, #8, #12, #31, #77, and #201).

### Absence of IG Light Chain Restriction in CLL-Like Stereotyped-IGs

**Figure 5:**
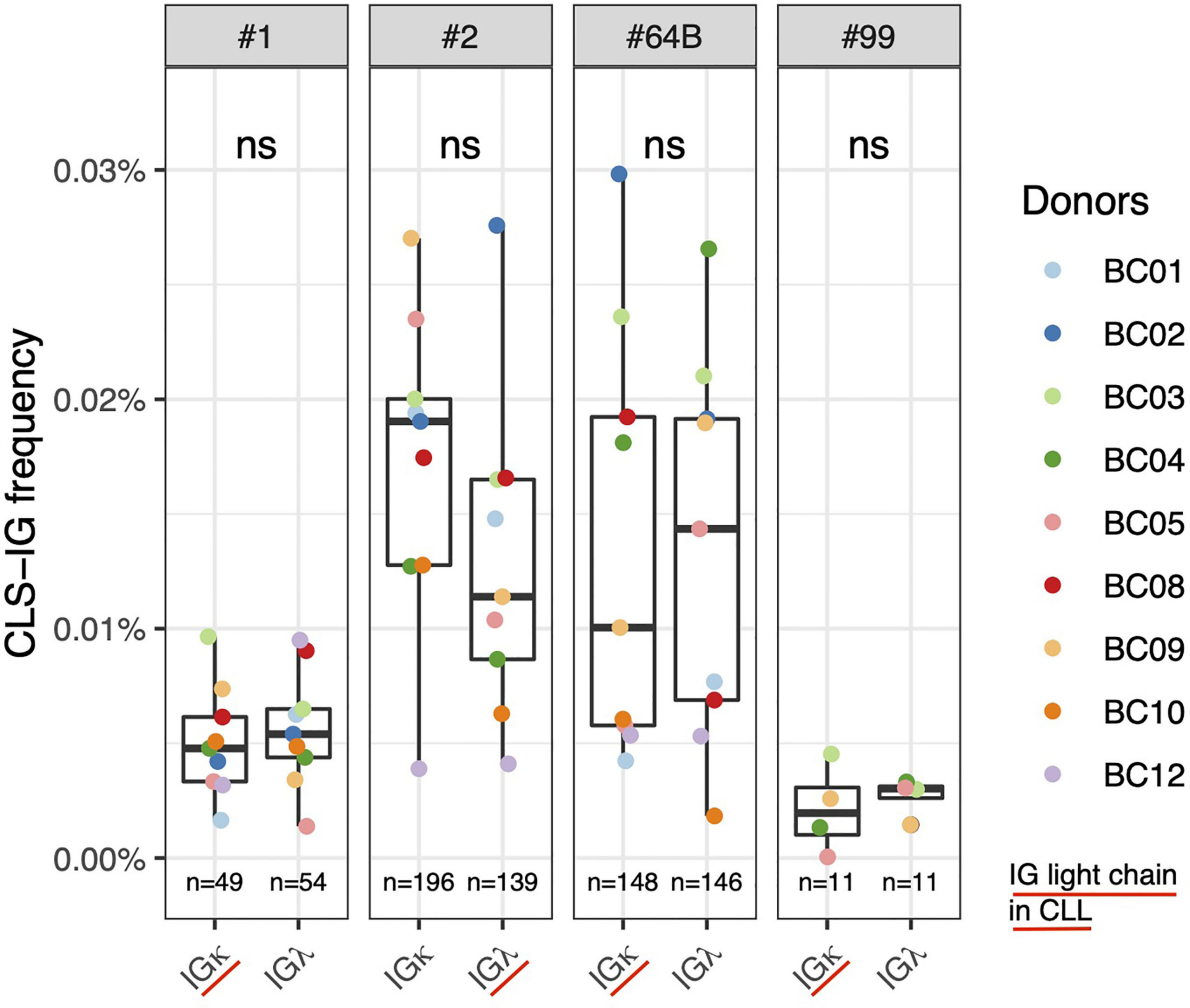
Frequency of individual CLS-IG in sequences from normal B cells expressing either IGκ and IGλ chains in CLL stereotyped subsets with reported IG light chainIGL bias (6, 12). Paired Wilcoxon test was performed. ns, not significant.

Many CLL clones with stereotyped receptors have restricted IG light chain usage (27, 28). This is particularly evident in leukemic clones utilizing subsets #1, #2, #4, #6, #8, #64b, and #99 (6, 12). We investigated whether CLS-IG from normal B cells showed light chain restriction. As each B-subset was fractionated starting from pre-sorted IGκ^+^ or IGλ^+^ B-cells and sequenced independently, we could obtain the CLS-IG distribution in both IGκ and IGλ-bearing cells. Among the CLL subsets for which clear evidence of IG light chain restriction is present in CLL clones, sufficient sequences for an informative analysis were obtained from subsets #1, #2, #64b, and #9 (**Figure 5**). There was no statistically significant difference in the IG light chain association for any CLS-IG analyzed. Only CLS-IG of subset #2 showed a trend indicating enrichment in IGκ^+^ normal B cells; this contrasts with CLL clones of the same subset that consistently show a pairing with IGλ. The entire distribution of the IG light chain among CLS-IG stereotypes is reported in **Supplementary Figure S2**.

### Similar Utilization of Mutated and Unmutated IGHV Genes by CLL Clones and CLL- Like Stereotyped-IGs

**Figure 6:**
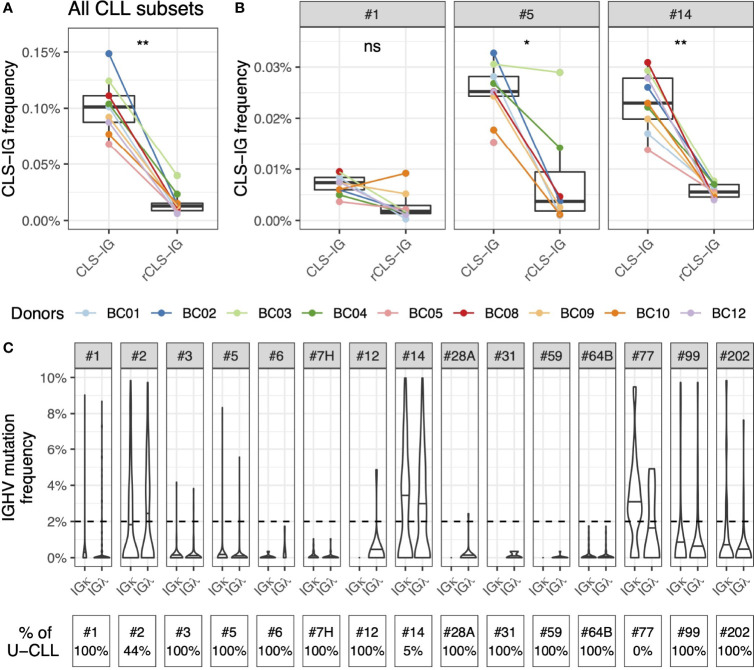
Similar utilization of IGHV mutated and unmutated genes by CLS-IG and CLL stereotypes. **(A)** CLS-IG and rCLS-IG frequency in normal B cells. **(B)** Frequency of CLS-IG and rCLS-IG of the #1, #5 and #14 CLL subsets in normal B cells. Paired Wilcoxon test was performed, only significant statistics are shown (*p ≤ 0.05, **p ≤ 0.01; ns, not significant). **(C)** Mutation pattern of the IGHV genes utilized by CLS-IG from normal B cells subdivided for the indicated CLL subsets; the horizontal lines indicate the median mutation for each subset. The dotted line indicates the 2% mutation threshold separating U and M sequences. The percentage of CLL stereotypes with unmutated IGHV in each CLL subset is shown at the bottom of the figure.

Most CLL stereotyped rearrangements are restricted to the utilization of U or M IGHV genes (6, 18). This characterizing feature is used by ARResT for CLL stereotyped subset assignment, meaning that a VH CDR3, potentially showing stereotyped features, will be excluded from a given subset if the IGHV gene, concomitantly utilized, fails to share the mutational status expected for that subset. Based on these considerations, we analyzed all the sequences from normal cells identified in this study to see whether there were restrictions to a specific mutational status for CLS-IG. To this end, we investigated whether VH CDR3 aa sequences, consistent with a CLL stereotyped subset, were present independently of the IGHV mutational status by extending the analysis to sequences with a reversed (r) mutational status compared to the core feature of the CLL subset (rCLS-IG) (see Methods). We observed that the “r” rearrangements were significantly less frequently classified as stereotyped than the native ones (**Figure 6A**). The same trend was observed for CLS-IG and rCLS-IG from individual CLL subsets when sufficient data were available for analysis (#1, #5, and #14, **Figure 6B**). The median mutation values of CLS-IG from the major CLL subsets followed a pattern like that of the corresponding CLL stereotyped-IGs (**Figure 6C**). For example, #1 was preferentially unmutated, #2 had around 2% mutations, and #14 was mutated. This pattern was similar for both the IGκ and IGλ B-cells.

### Identification of “Typical” and “Non-Typical” CLL-Like Stereotyped-IGs

**Figure 7:**
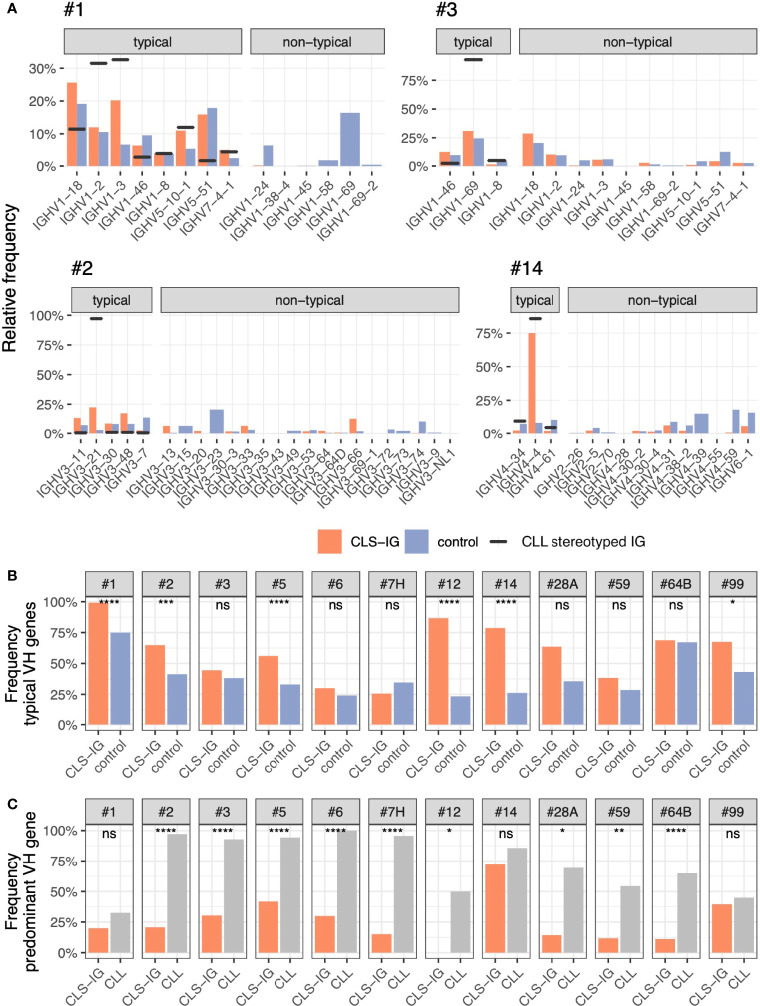
**(A)** Frequency of IGHV gene used in typical and non-typical CLS-IG (red). Blue bars indicate non-CLS-IG control sequences (i.e., sharing the same core features as the CLL subset in consideration - IGHV clan, IGHV mutational status, and VH CDR3 length). These rearrangements were used as controls. Black horizontal lines indicate the level of IGHV representation in the reference CLL cohort. The predominant IGHV gene was identified as the most represented within a CLL subset. **(B)** Frequency of typical IGHV genes in CLS-IG clonotypes compared to control sequences. The control sequences are the same reported in panel **(A) (C)** Frequency of the predominant IGHV gene observed in CLL stereotyped IGs compared to CLS-IGs usage of the same IGHV gene. Binomial test was performed, only significant statistics are shown (*p ≤ 0.05, **p ≤ 0.01, ***p ≤ 0.001, ****p ≤ 0.0001; ns, not significant).

One of the core features defining each CLL stereotyped subset is represented by the VH clan utilized, and, in each CLL subset, the rearranged IGHV genes often present a restriction at this level (6). This restriction ranges from about half the genes within a clan to a single gene (i.e., #4 - IGHV4-34, #8 - IGHV4-39). Again, all the CLS-IG sequences of this study were analyzed and classified as “typical” if expressing an IGHV gene of those found at least once in their respective CLL counterparts (6) and “non-typical” if expressing a different IGHV gene within the VH clan. For each CLL subset, the CLS-IG VH genes usage distribution was compared to control sequences from the same dataset. Control sequences were defined as sharing the core features of the CLL subset in consideration (6) (e.g., #1 - the use of a clan I VH gene, a U-IGHV gene, and a VH CDR3 length of 13 aa). The patterns observed varied among subsets (see **Figure 7**) and can be summarized in three broad categories as follows: i) CLS-IG falling almost exclusively in the typical category as in subset #1, where even though all rearrangements but one (1/379) were typical, the IGHV gene usage followed a distribution that was not consistent with that observed in either CLL or control non-CLS-IG sequences. ii) CLS-IG distributed in typical and non-typical categories with an IGHV gene distribution like non-CLS-IG control sequences and not presenting the CLL IGHV bias. Two examples of this condition were the CLS-IG sequences from subsets #2 and #3, which utilized several IGHV genes (with a frequency similar to that of non-CLS-IG control sequences) rather than the IGHV3-21 and the IGHV1-69 genes characterizing the #2 and #3 CLL subset, respectively. iii) CLS-IG showing the same bias toward the utilization of a specific IGHV gene marking the CLL stereotyped subset. An example is subset #14, where most CLS-IG expressed the IGHV4-4 gene like the CLL stereotyped-IG and, in this respect, were different from non-CLS-IG control sequences. When separating the sequences by the IG light chain used, some fluctuation in the IGHV gene used could be observed; nevertheless, the general pattern described above remained valid for sequences from both the IGκ and IGλ group (see **Supplementary Figure S3**). **Figures 7B, C** summarize some statistics of what is shown in **Figure 7A**. In half of the informative CLL subsets (#1, #2, #5, #7H, #12, #14, and #99), we observed a significantly higher representation of typical IGHV genes in CLS-IG compared to controls (**Figure 7B**). As for the predominant IGHV gene often noticed in each single CLL subset, we observed that this bias generally was not present in CLS-IG. Indeed nine out of the 12 CLS-IG did not show evidence for this condition (**Figure 7C**).

## Discussion

The identification of CLL clones’ stereotypical IG receptors indicates that different individuals share immunoglobulin rearrangements with these specific features. This aspect reminds the existence of identical CDR3 aa sequences between individuals, which defines the so-called public repertoire (10, 29). More recently, the definition of public repertoire has been made less stringent by including rearrangements with a high degree of similarity (30). In this respect, the public repertoire could enclose stereotypic rearrangements recognized in CLL clones.

The scope of this study was that of investigating whether, in healthy donors, CLL IG stereotyped sequences were present in the IG repertoire of any of the circulating B-subsets and to which extent. The donors analyzed did not have evidence of lymphocytosis, thus excluding the possibility of introducing biases due to some type of preleukemic stage, which may result in B-cell repertoire alterations (31). Blood samples were collected from donors with a median age of 58.8 (range 56-67), which is in the range of possible identification of clonotypes ascribable to CLL clones that became clinically evident later. Indeed, it has been reported that clonotypes attributable to the leukemic clone can be identified in the PB up to 16 years before CLL diagnosis (32). It is, however, unknown whether phenotypic changes were present in the PB B lymphocytes at this early stage. The age choice appears relevant for comparison with CLL, given that changes in B cell repertoire within single B-cell subsets may occur with advancing age and may influence the cell population in which leukemogenesis occurs. In addition, it has been reported that changes in the representation of certain CLS-IG can be observed in aging individuals (19).

Although CLL stereotyped receptors have been reported in healthy donors (18, 19, 33–35), our approach provides more details on their phenotypic features, considering, for instance, the expression of IG light chains and CD5 expression within B-subsets. The identification and characterization of CLL stereotyped IG in healthy donors could provide information on the cell(s) of origin of CLL. Attempts to identify a specific B-subset as a compartment of origin for CLL have been unsatisfying. Here we attempt to lay the ground for a better understanding of the CLL cell of origin.

Consistent with previous reports (20, 21), our data show a significantly higher presence of CLS-IG in bulk CD5^+^ compared to bulk CD5^-^ B cells. Nevertheless, analysis of U- and M-IGHV gene rearrangements within CD5^+^ and CD5^-^ B cells highlighted no enrichment of CLS-IG in CD5^+^ compared to CD5^-^ B cells within each mutational category (**Figure 2B**). Instead, CLS-IGs were enriched in U-IGHV genes regardless of being expressed by CD5^+^ or CD5^-^ B cells.

CLS-IG representation was also investigated in B-subset and their respective CD5^+^ and CD5^-^ fractions. The analysis showed a higher CLS-IG percentage in TR and N than in MZ and SM B-subset (**Figure 3A**), a finding in line with a previous study from our group on splenic CLS-IG carrying the IGHV-1 family genes (20). Since TR and N cells utilize almost exclusively U-IGHV rearrangements (**Figure 3C**), the higher representation of CLS-IG in these cell fractions is likely related to the predominance of U-IGHV rearrangements. The analysis of CD5^+^ and CD5^-^ cells from each B-subset separately showed no dominant presence of CLS-IG in any of the cell fractions studied (**Figure 3B**). However, MZ shows an evident trend where CD5^+^ have more CLS-IG than CD5^-^. It must be noticed that MZ presents a considerable amount of U-IGs concentrated in the CD5+ fraction (**Figure 3C**). It must be mentioned that because CD5^+^ B cells are scarce in predominantly mutated B cells subsets, a limited number of IG rearrangements were obtained in some CD5^+^ B-cell fractions (i.e., MO, SM, DN), impairing the possibility of statistical analysis (see **Supplementary Table S2** for details). Altogether, our data do not present evidence of a phenotypically distinct B-subset with a significant accumulation of CLS-IG. Their predominance in CD5^+^ N and TR B cells depends upon the higher presence of U-IG rearrangements in these B-cell fractions.

The relative distribution of CLL subsets in normal B cells did not follow that typically reported for CLL (**Figure 4B**). For example, CLL subsets #4 and #8, relatively frequent among the CLL major subsets, were rarely (or never) identified. A preferential representation of individual CLS-IG in the IGκ or IGλ expressing B-subset was not observed. In contrast, specific CLL subsets (e.g., #1, #2, #4, #6, #8, #64b, and #99) show, in CLL, marked IG-light chain use restrictions (6, 12, 36). It might be worth mentioning that CLS-IG #2 showed a trend indicating a higher representation in IGκ^+^ normal B-cells. This would be compatible with the observation that, in some instances, CLL clones belonging to subset #2 show functional rearrangements of the IGκ light chain inactivated with a Kappa Deleting Element (37). Thus IG-receptor editing may operate in this apparently large proportion of CLS-IG subset #2/IGκ^+^ B cells. These observations indicate no major structural constraints contributing to certain IGH-IG light chain pairing in the CLS-IG of a normal B-cell repertoire. This is in keeping with what has been reported about the possibility of CLL subset #2 IGHV rearrangement to pair with “non-native” (e.g., other than IGLV3.21 encoded IGλ light chain) IG light chains (38).

The IGHV genes used by CLS-IG rearrangements within each CLL subset showed a heterogeneous pattern. Their overall utilization was not always as restricted as CLL stereotyped IG. For instance, the IGHV1-69 gene was virtually absent in CLS-IG from CLL subset #1, even though this gene is one of the IGHV1 genes highly represented in control IGs. CLS-IG in CLL subsets #1 and #3 exemplify the absence of IGHV selection, whereas CLS-IG in CLL subset #14 acts closely to what is observed in CLL with a highly prevalent representation of the IGHV4-4 gene. This indicates that specific VH CDR3 sequences may have a non-random association with IGHV genes within a VH clan, possibly related to restrictions during IGHV-IGHD-IGHJ rearrangements and/or positive and negative selection in ontogenesis or the course of early antigenic challenges. The absence of IGκ and IGλ restriction for each of these CLS-IG subsets points out, at this stage, negligible participation of the IG light chains. Altogether, the leukemogenic process likely involves a further selection of IGHV genes and the IG light chains in a CLL stereotype-specific manner.

The mutational status of CLS-IG deserves particular comment. When the CLS-IG analysis was extended by removing the IGHV mutational status as a prerequisite for classification (see Methods), the representation of CLS-IG mainly followed the mutational status characterizing the original CLL clones. For instance, subset #1 (always unmutated in CLL) was identified predominantly in the U-IG repertoire of normal donors and the majority of subset #14 CLS-IG (mutated in CLL) were recognized in the M-IG repertoire. Likewise, subset #2 CLS-IG were found in the U and M CLS-IG repertoire, as observed in the CLL cohorts. The observation that the mutational status of CLS-IG parallels that of CLL IG stereotypes suggests that specific IG rearrangements could influence clonal function, e.g., by limiting the generation of a post-germinal center progeny (CLL subset #1) or by determining an accumulation of memory B cells (CLL subset #14).

Thus, single CLS-IG in recirculating B cell has only marginally superimposable features compared to those encountered in CLL clones and each one appears to have its characteristics. For instance, subsets #4 and #8 are substantially absent in the CLG-IG repertoire; subset #1 CLS-IG shows the utilization of IGHV genes closer to CLL stereotypes, whereas that of CLS-IG, subset #3 is more random. It can be presumed that the trajectory determining the emergence of CLL clones is very heterogeneous.

The above data demonstrate that CLS-IG detected in peripheral B cells from donors with no evidence of peripheral lymphocytosis have different features than those typically identified in leukemic clones, suggesting the shaping of CLL BCR repertoire and the emergence of the leukemic clones is dictated by numerous selecting factors. A recent study (32) showed that skewing of the B-cell repertoire is observable in some clusters before the clinical presentation of CLL. Thus, subjects in a pre-leukemic phase or predisposed to developing CLL are likely to have a different condition than the donors analyzed here. The observations that CLS-IGs are not enriched in PB derived B cells with a defined phenotype can be interpreted in different ways: 1) CLL cells originate from non-circulating B cells residing in solid lymphoid tissue, 2) CLL cells originate from B cells with a different phenotype than the ones explored in this study, 3) CLL cells originate from B cells without a defined immunophenotype. The above interpretations may not be mutually exclusive. In addition, it is possible that the leukemogenic process can be accompanied by immunophenotypic changes that encompass an elevated expression of CD5 typically observed in CLL and MBL cells and presumably the pre-monoclonal B-cell lymphocytosis described by Kolijn, P. et al. (32)

In this context, B-cell immune-genotype appears to be a relevant factor in the quest to identify the CLL clones’ cell of origin, adding additional elements useful for understanding CLL emergence routes.

## Data Availability Statement

The datasets presented in this study can be found in online repositories. The names of the repository/repositories and accession number(s) can be found below: https://www.ncbi.nlm.nih.gov/genbank/, PRJNA807871.

## Author Contributions

DB designed the study, designed the library preparation protocol, analyzed data and wrote the manuscript. MoC performed FACS Sorting, prepared the library, sequenced and analyzed the data and wrote the manuscript. DR designed and performed FACS Sorting. SM and RM processed biological samples and prepared the library. GU and VA collected samples. LA, SV, and AM performed data and statistical analysis. AN, FM, and GC supervised research. MaC and FG prepared the figures. MF and FF designed the study and wrote the paper. All authors read and approved the final manuscript.

## Funding

This work was supported by: Associazione Italiana Ricerca sul Cancro (AIRC) ID.15426 (to FF); AIRC and Fondazione CaRiCal co-financed Multi-Unit Regional Grant 2014 n.16695 (to FM); Italian Ministry of Health 5 × 1000 funds [2014 ([to GC), 2015 (to FF), 2016 (to FF and GC), Ricerca Corrente 2016 (to FF and GC); Gilead fellowship program 2016 (MC) and 2017 (GC); Associazione Italiana Ricerca sul Cancro (AIRC) Grant 5 x 1000 n. 9980 (to FM and AN); AIRC IG 24365 to AN; Associazione Italiana Leucemie, Cosenza (to FF). The primers for the determination of IGHV repertoire were kindly provided by TIB Molbiol srl (Genoa, Italy). AM received funding from the European Union’s Horizon 2020 Research and Innovation Programme under the Marie Skłodowska-Curie grant agreement No. 101023721.

## Conflict of Interest

The authors declare that the research was conducted in the absence of any commercial or financial relationships that could be construed as a potential conflict of interest.

## Publisher’s Note

All claims expressed in this article are solely those of the authors and do not necessarily represent those of their affiliated organizations, or those of the publisher, the editors and the reviewers. Any product that may be evaluated in this article, or claim that may be made by its manufacturer, is not guaranteed or endorsed by the publisher.

## Supplementary Material

Supplementary Figure 1 FACS sorting gating strategy **(A)** Rosettesep enriched B cells were depleted of IgA^+^ and aqua dead cells in all of the sorting procedures **(B)** four-way pre-sort with yield setting to separate enriched viable B cells into IGκ^+^/CD5^+^, IGκ^+^/CD5^-^, IGλ^+^/CD5^+^ and IGλ^+^/CD5^-^ B cells **(C)** IGκ^+^/CD5^-^ B cells subset (here shown as representative of all the other above subsets) was further sorted by gating CD24^-^CD38^high^ B cells to exclude plasmablasts and separated into CD24^high^CD38^high^ transitional (TR). Gating CD38^-/+^ B cells were then separated based upon IgD and CD27 expression markers and sorted as IgD^++^CD27^-^ naive (N), IgD^low^CD27^+^ marginal zone-like (MZ), IgD^-^CD27^-^ double negative (DN) B cells. IgD^-^CD27^+^ memory B cells were gated to isolate IgM^+^IgD^-^CD27^+^ IgM-only memory (MO) and IgM^-^IgD^-^CD27+ switch-memory (SM) B cells.

Supplementary Figure 2 Frequency of individual CLS-IG in sequences from normal B cells expressing either IGκ and IGλ chains in CLL stereotyped subsets.

Supplementary Figure 3 Frequency of IGHV genes used in typical and non-typical CLS-IG (red). Blue bars indicate non-CLS-IG control sequences (i.e., sharing the same core features as the CLL subset in consideration - IGHV clan, IGHV mutational status, and VH CDR3 length). These rearrangements were used as controls. Black horizontal lines indicate the level of IGHV representation in the reference CLL cohort.
