# Supplementary figures and images for: Characterizing Features of Human Circulating B Cells Carrying CLL-Like Stereotyped Immunoglobulin Rearrangements
Source: Front Oncol. 2022 Jun 23;12:894419. doi: 10.3389/fonc.2022.894419 (PMC9275393; doi:10.3389/fonc.2022.894419)

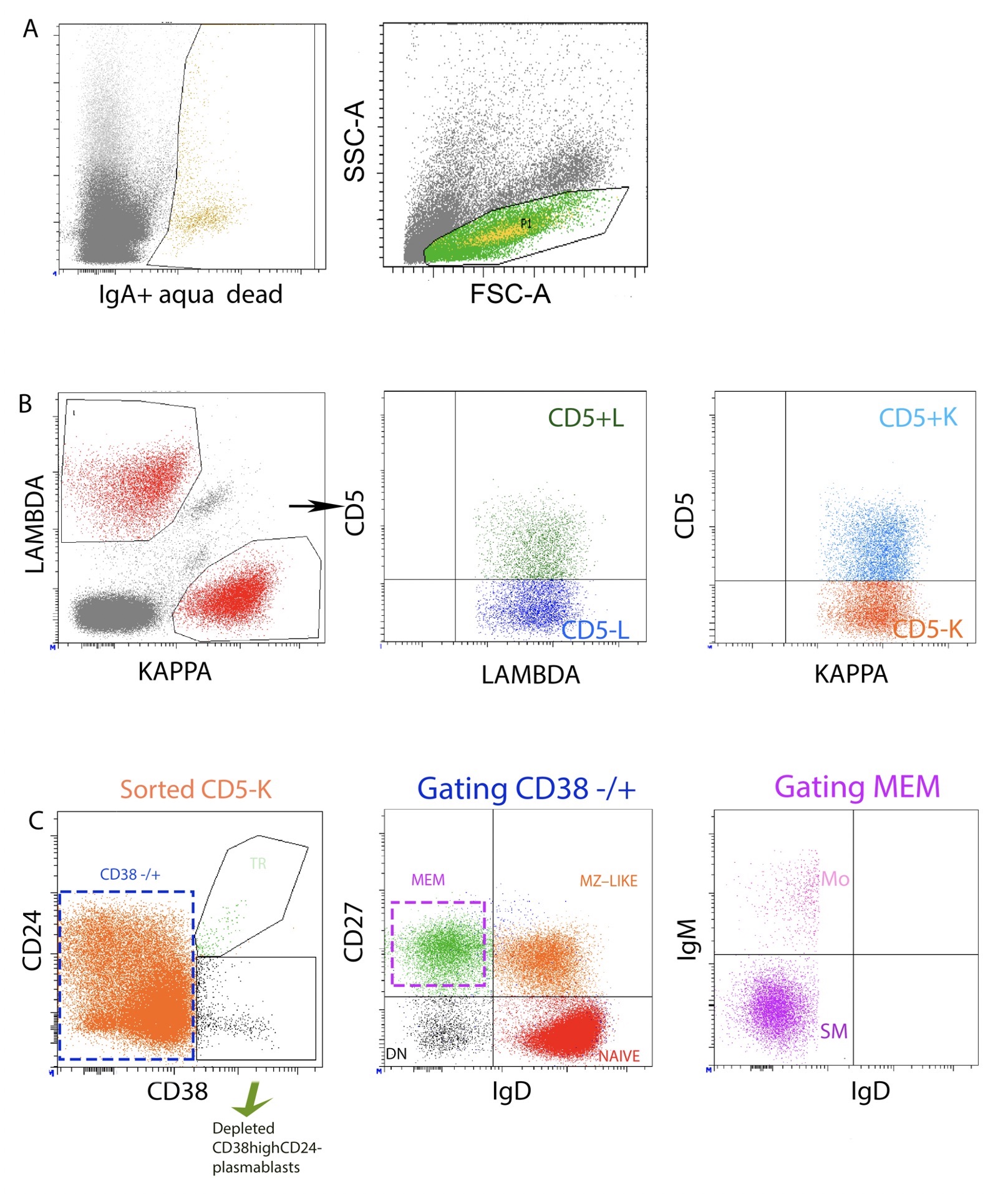

Supplement: Supplementary Figure 1 — FACS sorting gating strategy (A) Rosettesep enriched B cells were depleted of IgA+ and aqua dead cells in all of the sorting procedures (B) four-way pre-sort with yield setting to separate enriched viable B cells into IGκ+/CD5+, IGκ+/CD5-, IGλ+/CD5+ and IGλ+/CD5- B cells (C) IGκ+/CD5- B cells subset (here shown as representative of all the other above subsets) was further sorted by gating CD24-CD38high B cells to exclude plasmablasts and separated into CD24highCD38high transitional (TR). Gating CD38-/+ B cells were then separated based upon IgD and CD27 expression markers and sorted as IgD++CD27- naive (N), IgDlowCD27+ marginal zone-like (MZ), IgD-CD27- double negative (DN) B cells. IgD-CD27+ memory B cells were gated to isolate IgM+IgD-CD27+ IgM-only memory (MO) and IgM-IgD-CD27+ switch-memory (SM) B cells. [file Image_1.jpeg]

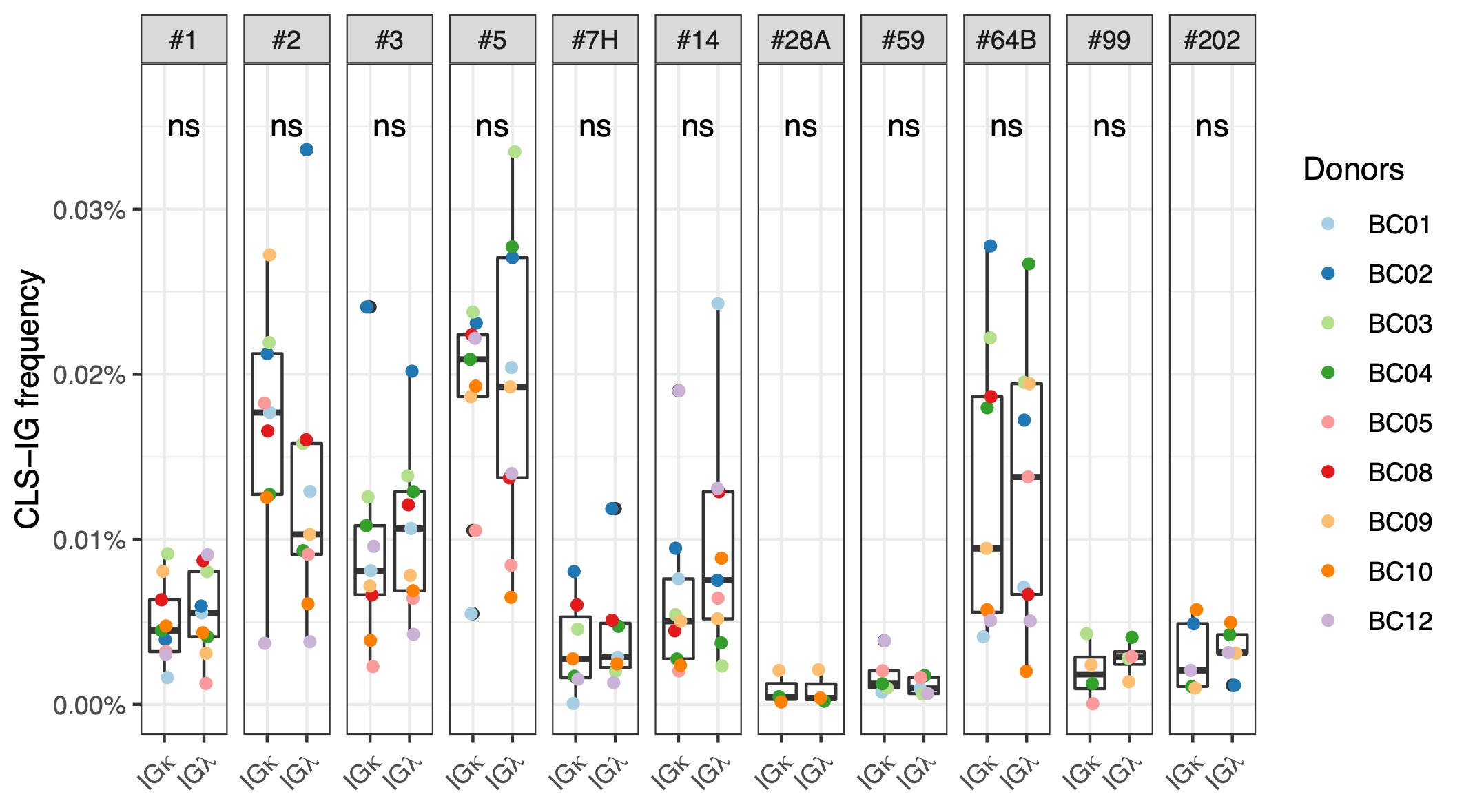

Supplement: Supplementary Figure 2 — Frequency of individual CLS-IG in sequences from normal B cells expressing either IGκ and IGλ chains in CLL stereotyped subsets. [file Image_2.jpeg]

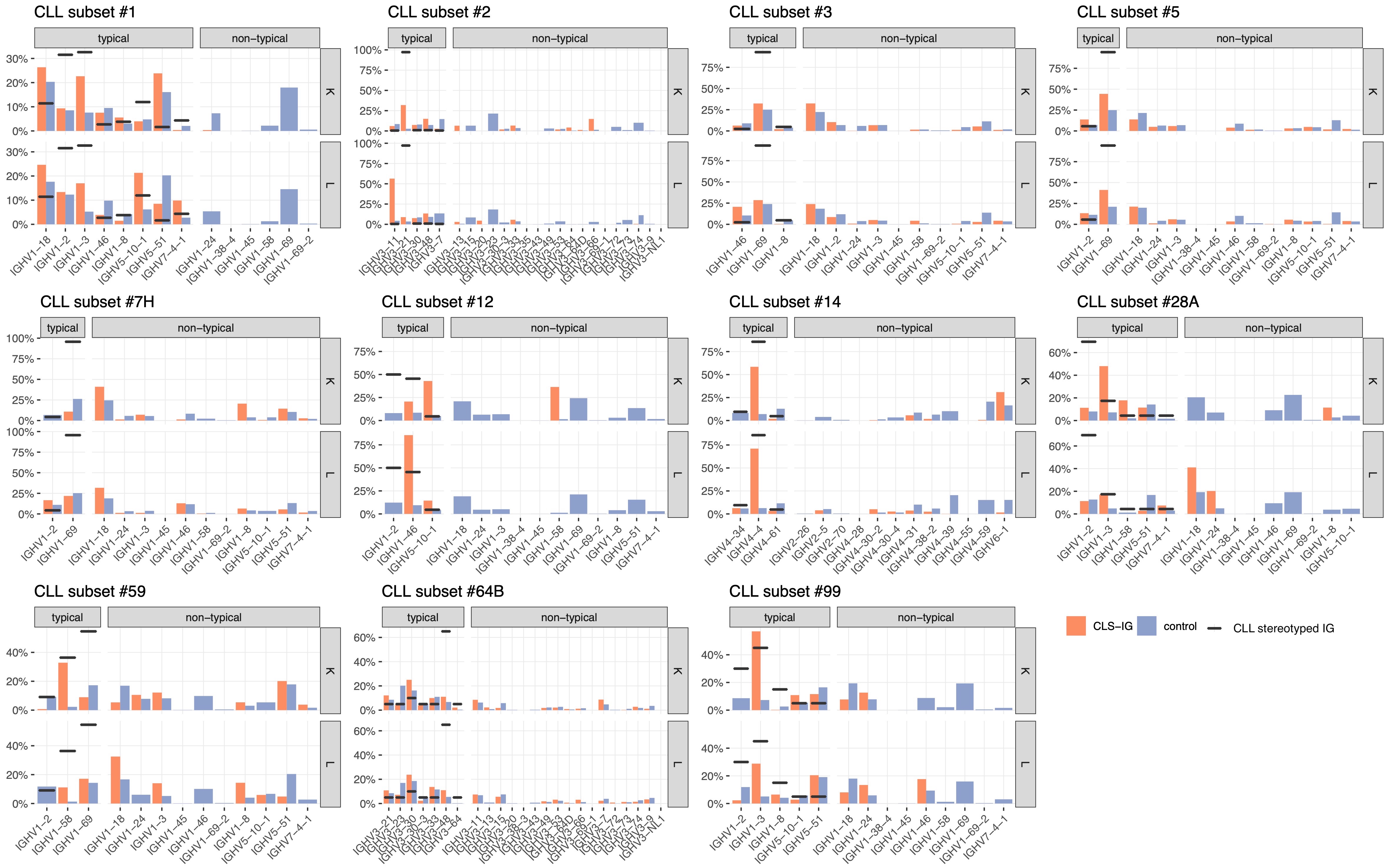

Supplement: Supplementary Figure 3 — Frequency of IGHV genes used in typical and non-typical CLS-IG (red). Blue bars indicate non-CLS-IG control sequences (i.e., sharing the same core features as the CLL subset in consideration - IGHV clan, IGHV mutational status, and VH CDR3 length). These rearrangements were used as controls. Black horizontal lines indicate the level of IGHV representation in the reference CLL cohort. [file Image_3.jpeg]
